# Mid-term results of impaction bone grafting in tibial bone defects in complex primary knee arthroplasty for severe varus deformity

**DOI:** 10.1051/sicotj/2018056

**Published:** 2019-01-14

**Authors:** Yashwant Singh Tanwar, Yatinder Kharbanda, Harsh Bhargava, Kulbhushan Attri, Anoop Bandil

**Affiliations:** 1 Department of Orthopedics, Apollo Hospitals Sarita Vihar Delhi 110076 India

**Keywords:** Primary knee arthroplasty, Tibial bone defect, Varus deformity, Impaction bone grafting

## Abstract

*Introduction*: Bone defects are a challenging problem encountered occasionally during primary knee arthroplasty. These defects should be meticulously addressed so as to avoid malalignment and premature loosening and failure. Out of the many options available to deal with these defects, impaction bone grafting provides a more biological solution, which is especially important in case of primary knees.

*Materials and methods*: A retrospective analysis was done and patients with severe varus deformity of more than 20 degrees who had undergone primary knee arthroplasty with impaction bone grafting of the tibial condyle defect were followed up.

*Results*: Between 2008 and 2014, out of the 1124 patients who underwent primary total knee arthroplasty, only 26 knees in 23 patients met the inclusion criteria. The amount of varus deformity ranged from 20 to 35 degrees. Follow-up ranged from 3 to 8 years with an average of 6 years. The average pre-operative Knee Society Score (KSS) and Western Ontario McMaster Universities (WOMAC) score were 24.2 and 78, respectively. There were significant improvements in the post-op scores, with the average KSS being 90.2 and the WOMAC being 38.

*Conclusion*: Impaction bone grafting provides an invaluable option to the orthopedic surgeon for managing bone defects, especially in case of primary knee arthroplasty as it reconstitutes the bone stock.

## Introduction

Bone defects are commonly encountered during revision knee arthroplasty and occasionally during primary surgeries. Most of the defects encountered during primary knee arthroplasty are minor ones and involve the tibia. The commonest location of these defects is the posteromedial corner of the tibia, as varus and flexion deformities are more common. Bone defects hinder correct implant alignment and reduce the prosthesis–host bone contact area, thereby reducing implant stability. Addressing bone defects adequately is therefore important for the long-term survivorship of the implant.

**Figure 1:**
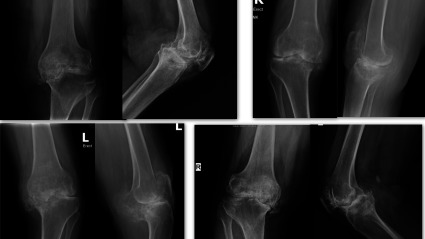
Pre-operative X-rays of patients with severe varus deformity and extensive bone loss from posteromedial condyle.

In developing countries, patients often present late with large bony defects similar to those encountered during revision surgery (Figure 1). There are a variety of surgical options available to deal with tibial bone defects, which include, lateralization of the tibial component, cementing alone or with screw reinforcement, metal augments, Impaction Bone Grafting (IBG), allografts, autografts, custom-made implants, and tantalum.

Insall classified bone defects as contained and uncontained. A contained defect has an intact cortical rim, whereas an uncontained defect has segmental bone loss with no remaining cortex. The size of the contained defect can be defined as small (<5 mm) or large (>5 mm). The size of the uncontained defect can be defined as small (<5 mm), intermediate (5–10 mm), or large (>10 mm) [1]. In 1991 Rand proposed a classification that considers the percentage extent of the defect into the tibial plateau or femoral condyle, distinguishing between four grades of increasing severity of the lesion (Table 1) [2].

**Table 1:** Rand classification of bone loss.

Type	Condylar involvement (%)	Depth (mm)
I (a/b)	<50	<5
II (a/b)	>50 to <70	5–10
III (a/b)	>70 to <90	>10
IV (a/b)	>90	>10

Note. (a) Intact peripheral rim. (b) Deficient peripheral rim.

The Anderson Orthopedic Research Institute (AORI) classification, although the one most commonly used, was described for revision cases [3].

We in the present series describe our experience with the use of IBG technique in managing uncontained tibial bone defects that involved 50% or less of the condylar surface area and were more than 10 mm in depth.

## Materials and methods

It was a retrospective study carried out at our institution. Inclusion criteria were:

1. Patients undergoing primary knee arthroplasty between 2008 and 2014.
2. Age >50 years.
3. Varus deformity >20 degrees.
4. Intact collateral ligaments.
5. Bone defects which involved 50% or less of the tibial condylar surface area and more than 10 mm in depth. The pre-op assessment gave only an estimate of the degree of bone loss. The final assessment of the degree of bone loss and the stability of the implant was done intra-operatively.
6. Patients in whom defect reconstruction was done using IBG and mesh and a stemmed tibial component was used. Patients in whom other methods of reconstruction were done were excluded.

## Surgical technique

**Figure 2:**
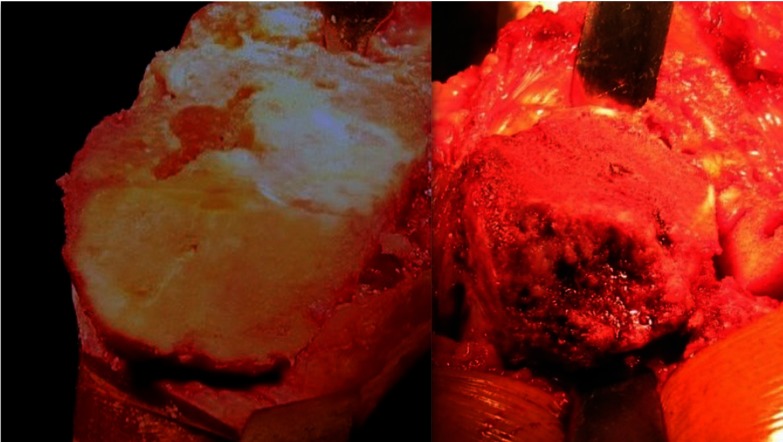
Bone loss from posteromedial tibial condyle after tibial cut.

**Figure 3:**
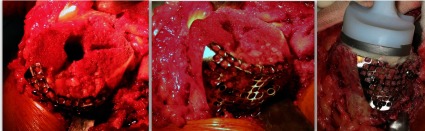
Intra-op images showing reconstruction of tibial defect with wire mesh and impacted bone graft.

A standard medial para-patellar approach was used in all cases. After removing the medial osteophytes, a standard postero-medial sub-periosteal release was performed in all cases as part of the exposure. The amount of bone defects was then quantified including the percentage of condylar area involved and the depth. A tibial cut with 0 degree posterior slope was given, with reference point being 8 mm below the normal lateral tibial plateau side (Figure 2). The amount of the bone that was cut was checked with the help of an angel’s wing, and the final cut was given removing minimal bone from the medial/defect side. Attention was then given to the femoral side, and a standard distal femoral cut of 8 mm with 5–7 degrees of valgus was given. Sizing of the distal femur was done using posterior referencing system; anterior, posterior, and chamfer cuts were completed with the help of an external jig. Flexion and extension gaps were then checked for symmetry and an additional posteromedial release was done if necessary. An appropriately sized tibial tray was kept over the resected tibial surface, and its rotation was checked with the help of an external alignment rod. Notch for the tibial keel was prepared. The tibial medullary cavity was now reamed to the largest possible extent for the stem component. A triangular wire mesh (Synthes TM) was used to convert the uncontained defect into a contained one, and it was fixed to the medial tibial plateau with the help of 3.5-mm screws. The defect base was perforated many times with a 2.5-mm drill bit and curetted. The bone graft obtained from the tibial and femoral cuts was morselized (5 mm) manually, and filled into the defect. Allograft was mixed if the autograft quantity was insufficient to fill the defect. Impaction of the graft was then carried out, which was the most important and time-consuming step in the process. Impaction of the graft was done with bone tamps and implant trials. While impacting the graft, the tibial keel punch was kept in place so as to achieve adequate graft impaction and also to prevent graft displacement into the tibial medullary cavity (Figure 3).

After achieving adequate impaction, the stemmed tibial and femoral trials were inserted. An adequate size of the tibial insert was selected, and the knee was checked for stability, range of motion, and patellar tracking. The final tibial component was cemented only in the metaphyseal region. While the shaft remained press-fit uncemented, the femoral component was cemented throughout in all the cases. Patellar replacement was not done in any of the cases. Active and active-assisted range of motion exercises of knee and full weight-bearing mobilization was started from the first day onward.

## Results

Between 2008 and 2014, 1124 patients underwent primary total knee arthroplasty. Out of these, only 26 knees in 23 patients met the inclusion criteria. Out of these, 10 were male and the rest female. The average age group of the patients was 63 years (range 56–73). Out of the 23 patients, 15 had primary osteoarthritis, 6 had inflammatory arthritis, and 2 had post-traumatic deformity. The amount of varus deformity ranged from 20 to 35 degrees (average being 25.6), and the average defect measured was 18 mm (range 12–34 mm). Follow-up ranged from 3 to 8 years with an average of 6 years. None of the patients had any loosening/subsidence or evidence of osteolysis till the last follow-up visit. The average flexion achieved was 102 degrees (range 95–120 degrees). The postoperative alignment was measured, which ranged from 2 degrees varus to 3 degrees valgus. The average pre-operative Knee Society Score (KSS) and Western Ontario McMaster Universities (WOMAC) score were 24.2 and 78, respectively. There were significant improvements in the post-op scores, with the average KSS being 90.2 and the WOMAC being 38.

Two patients had pes anserine bursitis probably owing to irritation from the wire mesh. Both cases settled with conservative management in the form of anti-inflammatory medicines, ice packs, and restriction of knee flexion for few days.

## Discussion

Extensive bone defects encountered in primary surgery may be due to many causes apart from late presentation, like previous condylar fractures with varus collapse, inflammatory arthritis, osteonecrosis of the condyles, presence of large subchondral cysts in osteoarthritis, and previous high tibial osteotomies. Usually the depth of bone defects on the tibial side does not exceed 8–10 mm. In such simple cases, the resection of the tibial plateau allows for the removal of most of the defects. However in more severe lesions, the tibial resection of more than 12 mm can lead to sacrifice of important ligamentous structures, which has been observed to considerably alter bone quality. The excessive tibial resection also leads to the impairment of the joint line level.

Of the many methods available to the surgeon for reconstructing a bone defect, only two have the aim of reconstituting the bone stock: impaction bone grafting and the use of structural allograft [4]. Bone grafts that do incorporate and remodel provide a more physiologic modulus of elasticity and trabecular pattern for load-bearing than do cement or metal implants.

Impaction bone grafting was first described in detail by Slooff et al. [5] in hip arthroplasty. The bone graft was impacted into the defect contained by a wire mesh followed by an insertion of an Acetabular cup into the pressurized cement. The use of morselized bone graft in conjunction with total knee replacement was first described in 1988 [6]. The use of IBG for uncontained defects using a wire mesh in revision knee arthroplasty was described by Lonner et al. [7]. Since then, many authors especially Lotke et al. have described the use of IBG in revision knee surgeries following its great success in hip replacement surgery [8, 9].

Various authors have described different techniques, varying in the methods and degree of morselization and compaction, and the use of long press-fit uncemented stems [10], long stems with hybrid cementing [11], long cemented stems [7, 8, 12], or the classic short stem design [13].

We in the present series used the hybrid cementing technique, wherein the cement is applied only to the metaphyseal portion of the tibial component and the stem is kept uncemented. Canal-filling cementless stems provide fixation by engaging in the diaphyseal bone distal to areas of metaphyseal bone loss. The advantage of these stems compared with cemented stems is that, should revision be necessary, the removal of a long cementless stem will be easier, with better retention of bone stock. Also cementless stems may better support restored bone stock by decreasing the proximal stress-shielding and improved loading of the graft. In a biomechanical study, Jazrawi et al. [14] reported a trend toward greater proximal stress-shielding, when cemented stems were compared with cementless stems [15].

Uncemented stems require good metaphyseal and diaphyseal bone quality and an endosteal diameter that permits engagement, therefore, relatively young patients undergoing primary arthroplasty are good candidates, as was the case in the present study. Whiteside and Bicalho in their study of 63 patients found morselized cancellous allografting of revision knees to be a reliable method when used in conjunction with firm rim seating and rigid intramedullary stem fixation. Two patients who required revision surgery had greatly improved bone stock so that new implants could be applied with minor additional grafting [16].

Toms et al. in their study of mechanical testing of impaction bone grafting in the tibia concluded that longer stems reduced permanent movement of the tray by almost 80%. Poor support of the rim increased movements of the tray by a factor of 2.6 and cyclical movement by a factor of 1.7, making short-stemmed trays vulnerable in particular. They also showed that rim support is an important factor in providing stability [17].

In another study, Toms et al. compared different methods of containment of tibial defects. They showed that the metal mesh was more flexible and did not restore the cortical thickness immediately, hence not providing much direct support. However, it still gave sufficient constraint to allow compaction of the morselized bone graft, and tray movements were within the range reported for primary uncemented knee prostheses. The study also showed that the variation in the size of the defect between 46% and 65% of the medial cortical rim had no significant effect on stability of the component when a wire mesh was used to contain the defect [18]. All these studies are consistent with our results as we did not encounter any cases with early loosening or graft lysis, and a good graft incorporation was achieved in all cases.

**Figure 4:**
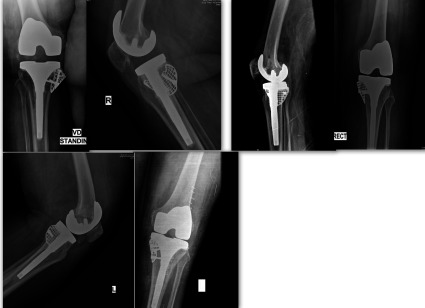
Post-operative X-rays with incorporation of impacted bone graft and well-aligned knee prosthesis.

Another possible advantage of mesh repair is that formation of a neocortex adjacent to the wire mesh is possible, as demonstrated in histological studies (Figure 4), a fact which becomes even more important in primary knees [16, 19].

The management of bone defects in primary surgery cannot follow the same principles as applied in revision cases as there are several important differences:

1. The important difference which the surgeon must keep in mind is that the patient might need a revision surgery later on; therefore, the modality of treatment chosen to treat the bone defect should be as biological as possible. This means that tantalum and other metal augments, which have recently become the most favored option for treating bone defects in revision surgery, should be used with caution in primary surgery. Although there have been reports of use of tantalum in primary arthroplasty [20], concerns still remain regarding such use.
2. In contrast to the soft cancellous host bone bed available for reconstruction in revision surgery, the host bed of primary knee is usually a hard sclerotic subchondral bone. This allows for a greater degree of compaction of the morselized graft. Studies have shown that the stability achieved is directly proportional to the degree of compaction [21, 22]. Although more vigorous impaction creates a more compacted graft bed with improved resistance to mechanical loading, it can also lead to intraoperative fractures, with fracture femur being the most common complication of the technique, when IBG is used on the femoral side during revision hip arthroplasty. A dense bone bed would also lessen the chances of intra-operative fracture.
3. Bone defects in most of the primary cases are confined only to the tibia as opposed to involving both the tibia and the femur in revision cases. This means that most of the defects can be filled with autogenous bone obtained from the cut itself, as opposed to a revision scenario, where minimal bone is available from cuts. A cancellous bone has better osteoconductive properties and hastens graft incorporation, whereas cortical allografts provide a more mechanical support [23]. The original technique of impaction bone grafting described by Slooff et al. involved the use of morselized cancellous bone [24]. We in the present series added morselized allografts to autogenous cancellous bone if the graft quantity was insufficient to deal with the defect.
4. Surgical time is not as important a constraint in primary surgery as it is in revision surgery, as the surgeon does not have to remove an existing implant. This seemingly non-essential factor becomes important when one considers that one of the drawbacks pointed out in the IBG technique is the time it requires for the impaction process. Therefore, the surgeon can devote adequate time to impacting the graft without compromising the quality.
5. Decreased biological potential of the host bone to incorporate the graft is usually not a factor in primary surgery as compared to revision surgery. This would theoretically lead to a better and rapid incorporation of the impacted graft.
6. Another advantage of the technique is that it allows the surgeon to fashion the graft to the defect at the time of surgery, rather than sacrificing the host bone to fit an allograft or an augment. This not only saves time but also preserves vital host bone stock. It is important, particularly in a younger patient, to minimize bone loss and to try to restore bone stock [25]. The addition of cortical support in the form of meshes makes the technique applicable to a range of scenarios involving various degrees of bone stock deficiency.

Despite the above advantages, there are, however, several drawbacks in our study, namely a small sample size, lack of control arm and comparative analysis, and a relatively short follow-up. This being a retrospective analysis, it inherits all the drawbacks of such a study design.

## Conclusion

Bone defects pose a challenging problem to the arthroplasty surgeon. There are different concerns while dealing with bone defects in primary and revision scenarios, which should be kept in mind. IBG provides the surgeon an opportunity to reconstitute bone stock, which is especially desirable in the relatively young patients undergoing primary knee surgery. IBG has been a time-tested modality in reconstructing acetabular bone defects and has been shown to have good long-term results. Its use in knee arthroplasty is relatively less explored, but it seems to be an attractive option due to many inherent advantages listed in the article. With a relatively simple and easily reproducible technique, further long-term studies with a larger number of patients are required to confirm the favorable results shown in this study.

## Conflict of interest

The authors declare that they have no conflict of interest.

## Funding

There is no funding source.

## Ethical approval

This article does not contain any studies with human participants or animals performed by any of the authors.
